# Surgical resection of mixed neuroendocrine-non-neuroendocrine neoplasm in the biliary system: a report of two cases

**DOI:** 10.1186/s40792-022-01386-w

**Published:** 2022-03-02

**Authors:** Ayano Tamaki, Yuma Tani, Hiroki Sato, Ryuichi Yoshida, Kazuya Yasui, Shigeru Horiguchi, Takashi Kuise, Yuzo Umeda, Kazuhiro Yoshida, Tomokazu Fuji, Kenjiro Kumano, Kosei Takagi, Takahito Yagi, Toshiyoshi Fujiwara

**Affiliations:** 1 Center for Graduate Medical Education, Okayama University Hospital, 2-5-1 Shikata-cho, Kita-ku, Okayama, 700-8558 Japan; 2 Department of Gastroenterological Surgery, Okayama University Hospital, 2-5-1 Shikata-cho, Kita-ku, Okayama, 700-8558 Japan; 3 Department of Gastroenterology and Hepatology, Okayama University Hospital, 2-5-1 Shikata-cho, Kita-ku, Okayama, 700-8558 Japan

**Keywords:** Mixed neuroendocrine-non-neuroendocrine neoplasm, Adjuvant chemotherapy, Ampulla of vater, Distal bile duct

## Abstract

**Background:**

Mixed neuroendocrine-non-neuroendocrine neoplasm (MINEN) is a rare disease and there is scarce literature on its diagnosis, treatment, and prognosis. We encountered two unusual cases of MINEN in the biliary tract, one in the ampulla of Vater and the other in the distal bile duct. In this report, we describe the clinical course of these two cases in detail.

**Case presentation:**

Case 1: A 69-year-old woman presented with a chief complaint of epigastric pain. When endoscopic sphincterotomy and retrograde biliary drainage were performed for gallstone pancreatitis, an ulcerated lesion was found in the ampulla of the Vater. Based on the biopsy results, the lesion was diagnosed as the ampulla of Vater carcinoma and subtotal stomach-preserving pancreatoduodenectomy (SSPPD) was performed. Postoperative histopathological examination revealed the coexistence of adenocarcinoma and neuroendocrine carcinoma components, consistent with the diagnosis of MINEN. In addition, lymph node metastasis was found on the dorsal side of the pancreas and the metastatic component was adenocarcinoma. Adjuvant chemotherapy with etoposide and cisplatin was administered for 6 months, and presently the patient is alive without recurrence 64 months after surgery. Case 2: A 79-year-old man presented with a chief complaint of anorexia. Cholangiography showed severe stenosis of the distal bile duct. A biopsy was conducted from the stenotic lesion and it revealed the lesion to be adenocarcinoma. A diagnosis of distal bile duct carcinoma was made, and SSPPD was performed. Histopathological examination revealed the coexistence of adenocarcinoma and neuroendocrine carcinoma components, and the tumor was confirmed as MINEN of the distal bile duct. No adjuvant chemotherapy was administered due to the poor performance status. 7 months later, the patient was found to have a liver metastasis.

**Conclusion:**

We experienced two valuable cases of biliary MINEN. To identify better treatments, it is important to consider the diversity of individual cases and to continue sharing a variety of cases with different presentations.

## Background

In 2010, the World Health Organization (WHO) defined tumors that were composed of adenocarcinoma and neuroendocrine carcinoma (NEC), with each component occupying > 30% of the lesion, as mixed adeno-neuroendocrine carcinoma (MANEC) [1]. According to the latest update in 2017, MANEC was renamed mixed neuroendocrine–non-neuroendocrine neoplasm (MINEN) [2]. Non-neuroendocrine components include not only adenocarcinoma, but also other types of cancers, such as squamous cell carcinoma and acinic cell carcinoma. In addition, the term "carcinoma" was replaced by "neoplasm" meaning that MINEN can include subtypes that have no malignant component. Therefore, the term "MINEN" now describes a broader disease spectrum, making it difficult to standardize the treatment of MINEN, and making it necessary to adopt treatment strategies case by case. Herein, we report two cases of the resection of MINEN in the biliary tract (one in the ampulla of Vater and the other in the distal bile duct). Biliary MINENs are relatively rare, accounting for 4–24% of digestive MINENs [3, 4]. Since preoperative diagnosis based on biopsy or image analysis is difficult, the optimal therapeutic strategy, especially in postoperative management, has not yet been clarified [5, 6]. We present a detailed clinical course of two MINEN cases, including their diagnosis, treatment, and prognosis.

## Case presentation

### Case 1

**Fig. 1:**
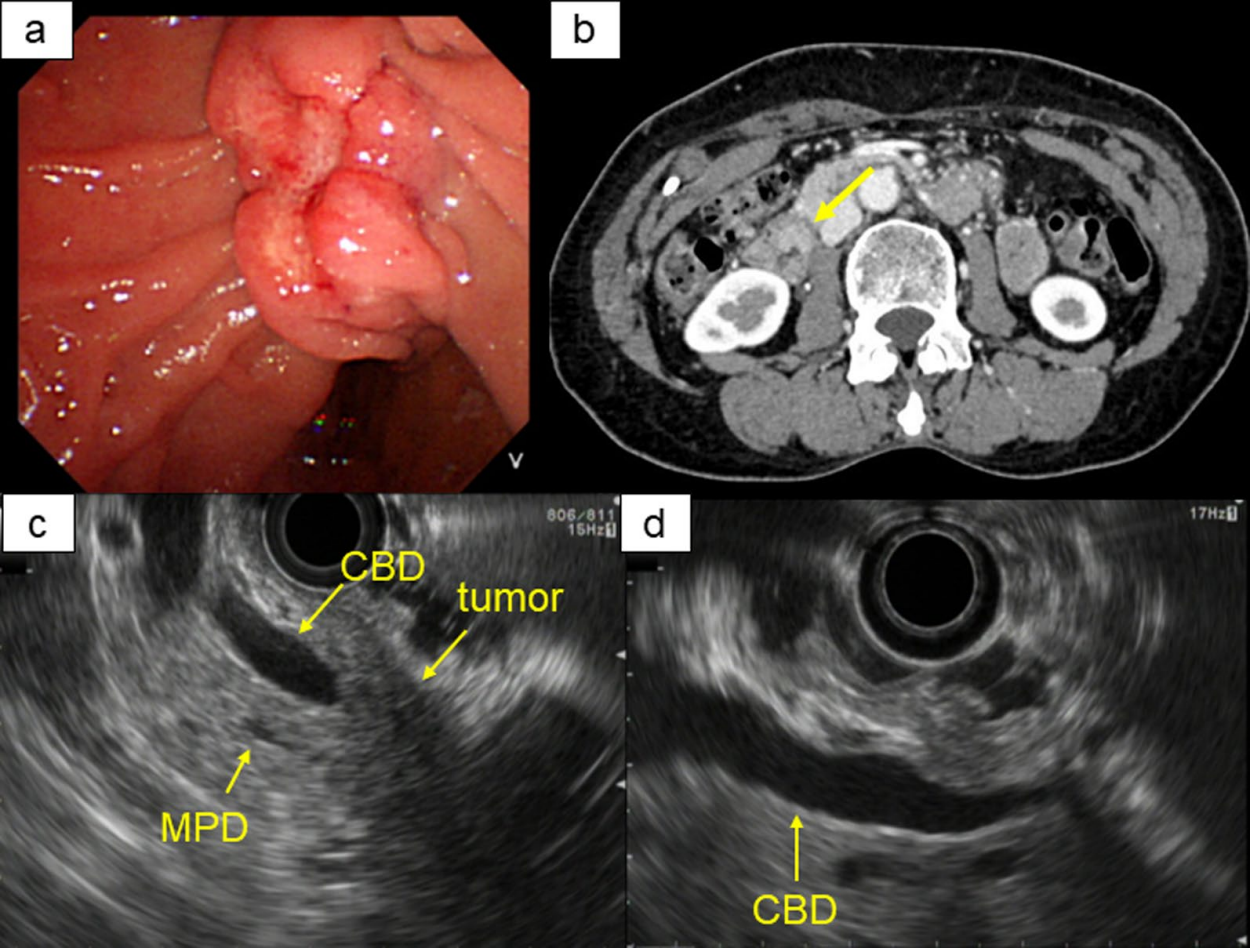
Preoperative examination. **a** Endoscopy revealed an ulcerative lesion in ampulla of Vater. **b** Contrast-enhanced CT showed a 1.5-cm enhancing nodule around papilla of Vater. **c**, **d** EUS suggested that the tumor may have invaded the pancreatic parenchyma. CT, computed tomography; EUS, endoscopic ultrasound

**Fig. 2:**
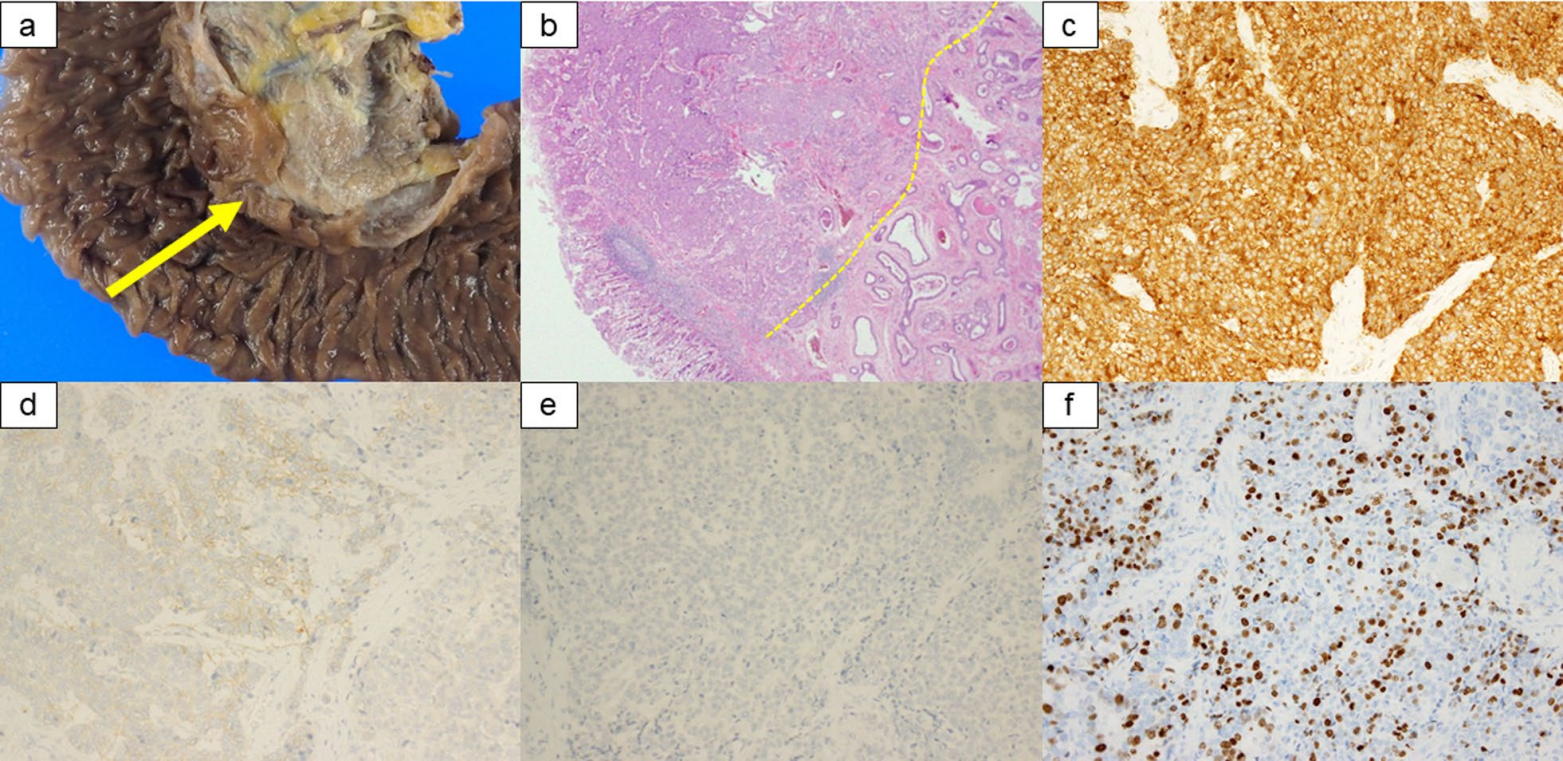
Macroscopic and microscopic findings of MINEN in ampulla of Vater. **a** Macroscopic finding of the tumor. **b** Microscopic finding showed the transitional part of neuroendocrine and non-neuroendocrine component. **c** Tumor cells were positive for synaptophysin. **d** Tumor cells were partially positive for CD56. **e** Tumor cells were negative for chromogranin A. **f** Tumor cells had a Ki-67 index > 20%. MINEN, mixed neuroendocrine–non-neuroendocrine neoplasm

A 69-year-old woman with a past medical history of hyperlipidemia, endometriosis (hysterectomy when she was 39 years old), and cholelithiasis reported to her family doctor with a chief complaint of epigastric pain. Laboratory evaluation revealed elevated inflammatory markers and serum amylase levels, and computed tomography (CT) scan findings revealed gallstone pancreatitis. Endoscopic sphincterotomy and retrograde biliary drainage were immediately performed, and upon examination, an ulcerative lesion was unexpectedly identified in the ampulla of Vater (Fig. 1a). Mucosal biopsy results indicated the pathological diagnosis as adenocarcinoma, and the clinical staging was evaluated after an improvement in pancreatitis was observed. Contrast-enhanced CT revealed a 1.5-cm enhancing nodule around the papilla of Vater (Fig. 1b) with no obvious distant metastases or swollen lymph nodes. Endoscopic ultrasound (EUS) showed a possible tumor invasion of the pancreatic parenchyma beyond the duodenal muscle layer (Fig. 1c, d). Endoscopic retrograde cholangiopancreatography (ERCP) revealed no findings suggestive of obvious invasion of the main pancreatic duct and common bile duct. Laboratory data were as follows: white blood count, 4380 cells/μL; hemoglobin level, 14.0 g/dL; platelet count, 25.7 × 10^4^ cells/μL; aspartate transaminase (AST), 17 IU/L; alanine aminotransferase (ALT), 13 IU/L; total bilirubin, 0.81 mg/dL; albumin, 4.4 g/dL; and creatinine, 0.65 mg/dL. The serum tumor markers, human carcinoembryonic antigen (CEA) and cancer antigen 19-9 (CA19-9), were within the normal range (1.02 ng/mL and 6.1 U/mL, respectively). Based on these results, the patient was diagnosed with clinical stage Ia (cT1bN0M0, Japanese Society of Hepato-Biliary-Pancreatic Surgery 7th edition) ampulla of Vater carcinoma. Subtotal stomach-preserving pancreatoduodenectomy (SSPPD) with modified Child's reconstruction and regional lymphadenectomy was performed. The surgical specimen showed a mixed-type tumor (12 × 6 mm, ulcerative-dominant type) (Fig. 2a). Histopathological examination revealed atypical cells with oval nuclei and increased chromatin that formed irregularly shaped ducts, indicating the existence of an adenocarcinoma component. In addition, atypical cells with salt-and-pepper chromatin with numerous mitoses (mitoses/10 high-power field > 20) showing solid-nest growth were also observed (Fig. 2b). Immunohistochemical examination of these cells showed that the tumor cells were positive for synaptophysin and partially positive for CD56, and negative for chromogranin A and CAM5.2. The cells had a Ki-67 index > 20% (Fig. 2c–f). These findings indicate the coexistence of poorly differentiated NEC components. Both components accounted for more than 30% of the tumor, which was consistent with the diagnostic criterion of MINEN. Lymph node metastasis was found in one of the lymph nodes on the dorsal side of the pancreas with the metastatic component being adenocarcinoma. The final pathological diagnosis was as follows: Adc, ulcerative predominant type, 12 × 6 mm, MINEN (large cell NEC > well), pT1b, int, INFb, ly2, v1, ne0, pN1 (1/16), pHM0, pPM0, pEM0, pPVX, pAX, R0 and pathological stage IA (T1N0M0, AJCC/UICC 8th edition). The postoperative course was uneventful, and the patient was discharged on the 17th postoperative day. Adjuvant chemotherapy with etoposide and cisplatin was administered for 6 months, and the patient is alive without recurrence 64 months after surgery.

### Case 2

**Fig. 3:**
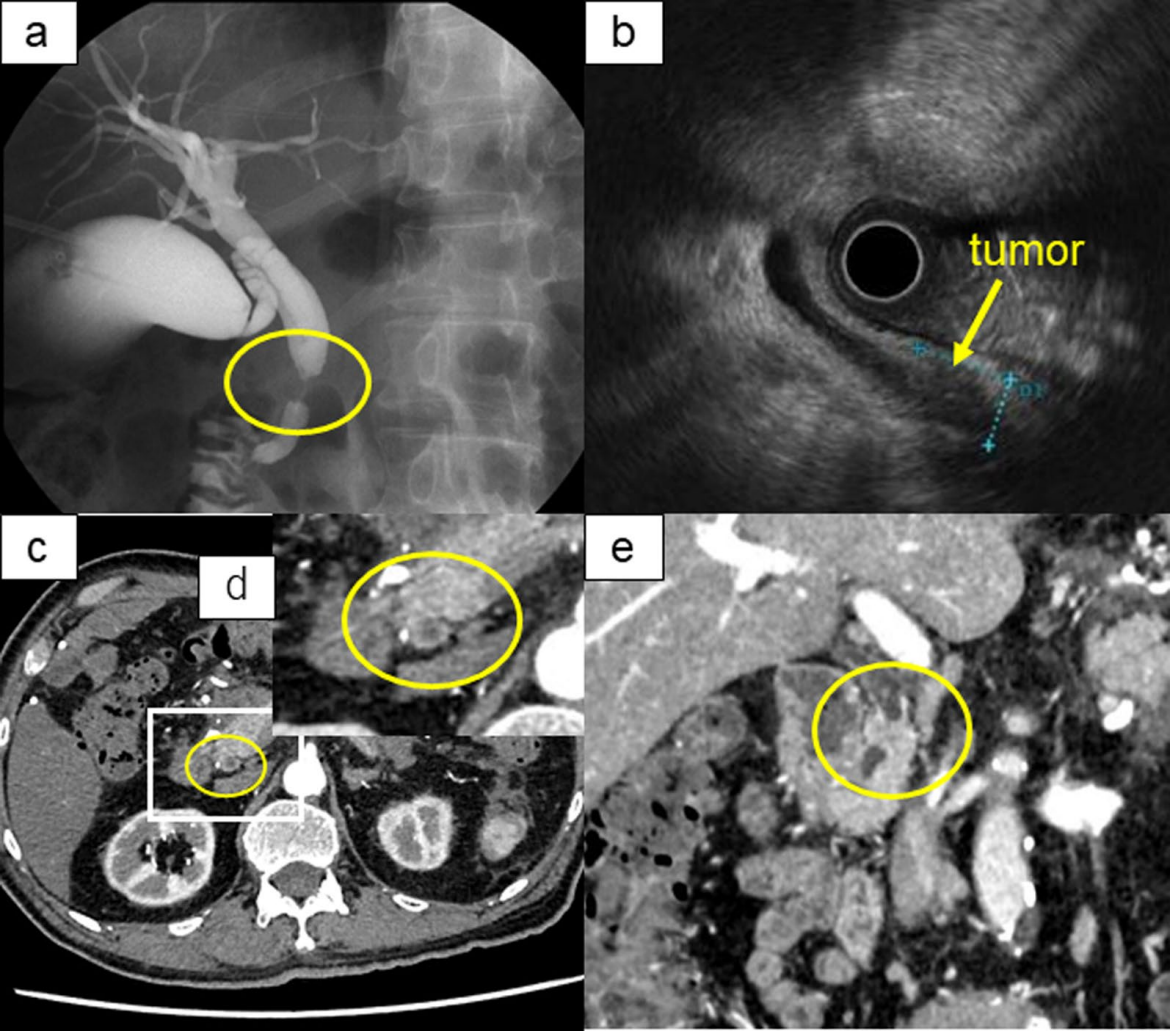
Preoperative examination. **a** Cholangiography showed severe stenosis of distal bile duct. **b** EUS showed a hypoechoic mass of 14 × 10 mm in the intrapancreatic bile duct and suggested no invasion of the pancreas and duodenum. **c**-**e** Contrast-enhanced CT showed circumferential wall thickening in the intrapancreatic bile duct. *CT* computed tomography; *EUS* endoscopic ultrasound

**Fig. 4:**
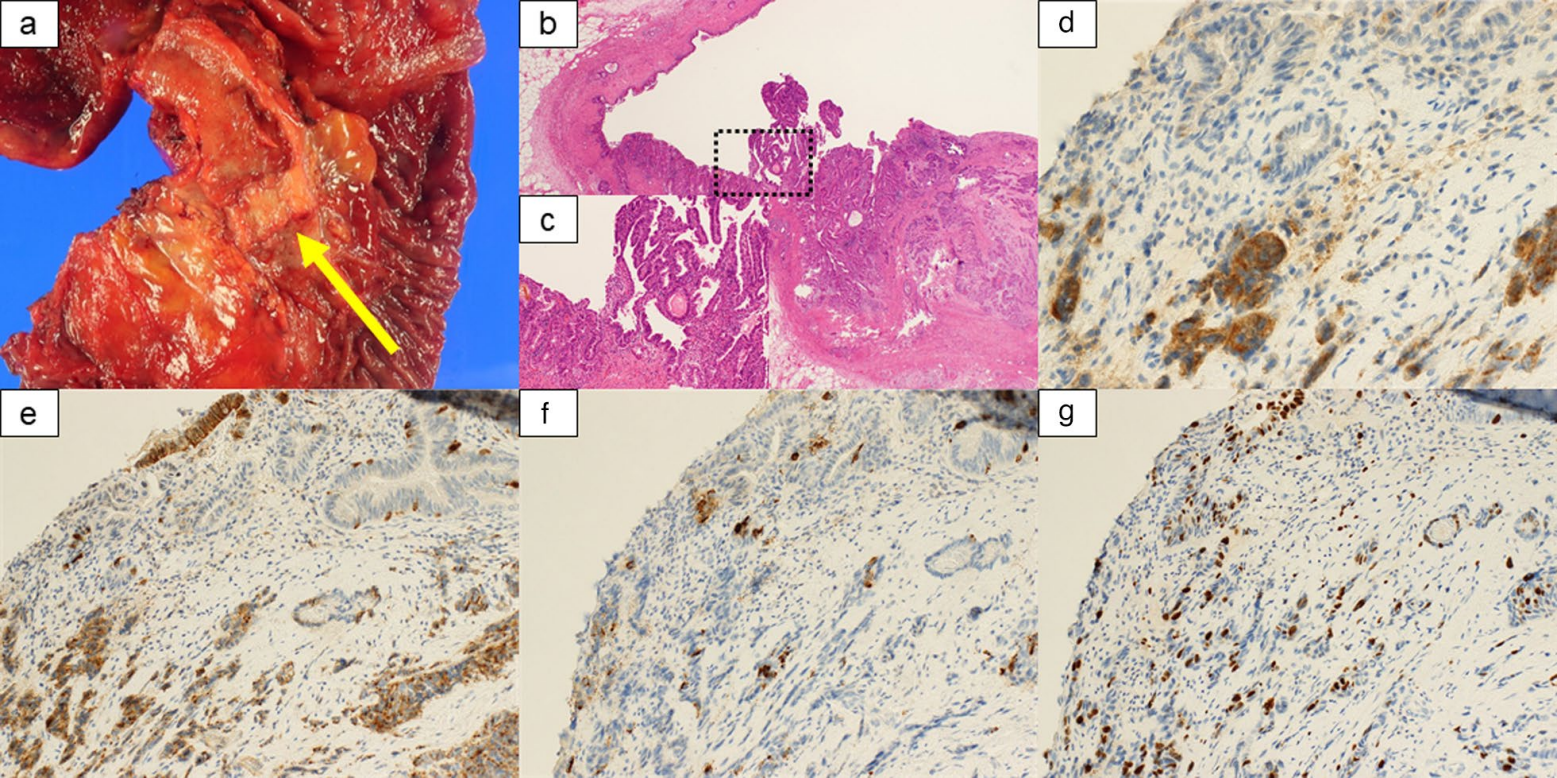
Macroscopic and microscopic findings of MINEN in distal bile duct. **a** Macroscopic findings of the mixed-type tumor (20 × 12 mm, nodular-infiltrating type). **b**, **c** Microscopic findings revealed coexisting tubular formation and solid-nest growth. **d**–**f** Tumor cells were partially positive for synaptophysin (**d**), CD56 (**e**), and chromogranin A (**f**). **g** Tumor cells had a Ki-67 index > 20%. MINEN, mixed neuroendocrine-non-neuroendocrine neoplasm

A 79-year-old man with a medical history of hyperlipidemia, hypertension, type II diabetes, benign prostatic hyperplasia, and stroke (he was on an anticoagulant therapy) reported to his family doctor with a chief complaint of anorexia. He was found to have elevated hepatobiliary enzyme levels: aspartate aminotransferase (AST), 594 IU/L; alanine aminotransferase (ALT), 355 IU/L; and gamma-glutamyl transpeptidase (γ-GTP),1900 IU/L. Ultrasonography revealed dilatation of the common bile duct and enlargement of the gallbladder. He underwent endoscopic retrograde biliary drainage and subsequently developed post-ERCP pancreatitis with extremely high levels of CRP (44.0 mg/L) which led to hospitalization for approximately 1 month. After an improvement in pancreatitis was observed, he was referred to our hospital and the cause of bile duct stenosis was investigated. Cholangiography showed severe stenosis of the distal bile duct and biliary cytology revealed adenocarcinoma (Fig. 3a). EUS findings showed a hypoechoic mass of 14 × 10 mm in the intrapancreatic bile duct and suggested no invasion to the pancreas and the duodenum (Fig. 3b). Contrast-enhanced CT showed circumferential wall thickening in the intrapancreatic bile duct (Fig. 3c–e) with no obvious distant metastasis. Laboratory data were as follows: white blood count, 4770 cells/μL; hemoglobin level, 14.3 g/dL; platelet count, 19.9 × 10^4^ cells/μL; AST, 19 IU/L; ALT, 21 IU/L; total bilirubin, 0.47 mg/dL; γ-GTP, 79 IU/L; albumin, 3.9 g/dL; and creatinine, 1.17 mg/dL. CEA and CA19-9 levels were 1.92 ng/ml and 43.8 U/ml, respectively. Laboratory findings indicated mild renal dysfunction and a slight elevation in CA19-9 serum levels. Based on these results, the patient was diagnosed with clinical stage IIa (cT2N0M0, Japanese Society of Hepato-Biliary-Pancreatic Surgery 7th edition) distal bile duct carcinoma. Left cervical and supraclavicular fossa lymph node swelling was detected by CT, and biopsy revealed a low-grade follicular lymphoma. Considering the degree of malignancy, the treatment of bile duct cancer was prioritized and SSPPD with modified Child’s reconstruction and regional lymphadenectomy was performed. Macroscopic examination of the resected specimen showed a nodular-infiltrating type tumor (20 × 12 mm) in the distal bile duct (Fig. 4a). Histopathological examination revealed that atypical cells with tubular formation and solid-nest growth were coexisting, and both components accounted for more than 30% of the tumor. Immunohistochemical examination of the section indicating solid-nest growth revealed the tumor cells to test positive for synaptophysin, CD56, and partially positive for chromogranin A. The growth had Ki-67 index > 20%, indicating the existence of the NEC component. Lymph node metastasis was found in one of the lymph nodes around the common hepatic artery, and its metastatic component was NEC (Fig. 4b–g). The final pathological diagnosis was as follows: Bd, circ, nodular-infiltrating type, 20 × 12 mm, MINEN (SCNEC > well), pT2(SI), pPV0, pA0, INFb, Ly0, V0, Pn1c, pN1 (1/9), pHM0, pPM0, pEM0, R0, and pathological stage IB (T2N0M0, AJCC/UICC 8th edition). The patients required a relatively long hospital stay because of his poor oral intake; however, he was discharged on the 33rd postoperative day. The patient is alive and being monitored without adjuvant chemotherapy in an outpatient setting; 7 months after surgery, the patient was found to have a liver metastasis.

## Discussion

Since the first report of a gastrointestinal tumor with an exocrine and a neuroendocrine component in 1924, several names have been used to describe tumors containing neuroendocrine and non-neuroendocrine components [7]. The term "MINEN" has been assigned to the tumor since 2017, and it comes to encompass a wide variety of tumors ranging from benign to malignant in nature, indicating the necessity of personalized assessment and treatment. In this article, we report two cases of resected biliary MINEN. Both patients underwent radical surgery based on the preoperative diagnosis of adenocarcinoma and were diagnosed with MINEN consisting of NEC and adenocarcinoma by postoperative pathological examination. The first patient with MINEN of the ampulla of Vater attained long-term survival through multidisciplinary treatment with surgery followed by adjuvant chemotherapy. While for the second patient with MINEN of the distal bile duct, it was decided not to administer adjuvant chemotherapy, despite being preferable, because of his poor performance status at the time of discharge.

Due to the few numbers of cases and their heterogeneity, the standard treatment for MINEN, especially involving adjuvant therapy, is still controversial. In their systematic review of biliary MINEN, Wen et al. reported that incomplete resection, Ki-67 index (≥ 50), tumor stage, NEC grade, and non-NEC grade were prognostic factors for poor overall survival, and that adjuvant chemoradiotherapy for those patients may contribute to better overall survival [8]. Yoshimachi et al. also reported in their literature review of ampullary MANEC that multidisciplinary treatment including chemotherapy may improve the prognosis of patients with MANEC [9]. Several reports suggested that because the NEC component was the main determinant factor for prognosis of patients with MINEN, the regimen of adjuvant chemotherapy for MINEN should target the NEC component [9–11]. In our case of ampullary MINEN, since lymph node metastasis was composed of adenocarcinoma, careful discussion was needed to decide which components should be targeted for adjuvant chemotherapy. The Japanese Guidelines for the Treatment of Pancreatic and Gastrointestinal Neuroendocrine Neoplasms recommend that postoperative adjuvant chemotherapy for pancreatic and gastrointestinal NEC should be a platinum-based combination therapy such as cisplatin in addition to irinotecan or cisplatin and etoposide, similar to the treatment of small cell carcinoma. Following this guideline, we eventually selected the regimen of etoposide and cisplatin that targets the NEC component with the expectation that cisplatin can also suppress the progression of adenocarcinoma, resulting in a favorable clinical course. On the other hand, Hatano et al. also reported an ampullary MINEN case with lymph node metastasis of adenocarcinoma. The patient was administered adjuvant chemotherapy with tegafur/gimeracil/oteracil (S-1) and was alive without recurrence for 8 months [12]. According to the literature review reported by Yoshimachi et al., seven out of 15 cases of ampullary MINEN received adjuvant chemotherapy (oxaliplatin-based combination chemotherapy; *n* = 5, S-1; *n* = 2) [9]. In addition, Wen et al. reported that 22 out of 51 patients with biliary MINEN received adjuvant chemotherapy, and 21 of 22 received platinum-based combination therapy [8]. Thus, platinum-based combination therapy is likely to be the mainstream adjuvant chemotherapy for MINEN following radical resection.

## Conclusion

In conclusion, we encountered two valuable cases of biliary MINEN. Considering the diversity of the individual cases, it is important to accumulate and share evidence-based experiences, which may lead to better outcomes in the future.

## Data Availability

Not applicable.

## Abbreviations

MINEN: Mixed neuroendocrine-non-neuroendocrine neoplasm
MANEC: Mixed adeno-neuroendocrine carcinoma
SSPPD: Subtotal stomach-preserving pancreatoduodenectomy
WHO: World Health Organization
CT: Computed tomography
EUS: Endoscopic ultrasound
ERCP: Endoscopic retrograde cholangiopancreatography
AST: Aspartate transaminase
ALT: Alanine aminotransferase
CEA: Carcinoembryonic antigen
CA19-9: Cancer antigen 19-9
NEC: Neuroendocrine carcinoma
